# The Dangers of "Comforters."

**Published:** 1912-08-10

**Authors:** 


### MATERNITY AND MOTHERING.

(Inquiries and Correspondence are invited.)

### The Dangers of "Comforters."

Not so very long ago the author of a pocket obstetric dictionary * included in his definitions the following succinct and sweeping denunciation: "Comforter.—A device for transferring dirt and disease germs into the mouth and stomach of an infant." And as a matter of fact there is accuracy as well as acerbity in this definition, which sums up the basis of the objections held by modern medical practitioners to the use of this common object.

These objections are becoming known to mothers of all classes; but a large proportion are apt to dismiss the warnings of their doctors with contemptuous disbelief. This attitude is easily understood: a distracted mother, endeavouring to get through the week's washing, whilst she keeps as watchful an eye as may be upon the peradventures of older children or upon the cooking of the family dinner; or darning clothes in the intervals of dyspepsia caused by drinking that black, stewed, astringent poison beloved by the democracy under the name of tea: such a mother may well be excused for taking any measures which promise to keep a squalling baby quiet for a few minutes at a time. She does not understand that in its frequent descents to the floor, the bone ring or rubber teat acquires a slight but deadly coating of microbe-laden dust and dirt: how should she understand the part thus played in the causation of diarrhœa and other digestive troubles of infants?

Difficult as it is to make this type of mother understand the pernicious nature of the "comforter," among the more educated classes the campaign against it is sometimes more difficult still. For with that half-knowledge of hygienic truths which is as yet all that most of the laity have attained, they meet one's objections with the reply that the offending instrument is "sterilised by the nurse" every morning! How long it remains sterile is, of course, a question they do not think of asking themselves.

### The Management of "Squallers."

Let it be accepted, then, as a proposition to be pressed upon mothers of all ages and all classes, that the "comforter," no matter of what substance composed, is a fertile source of digestive troubles among infants, because its usage results in the swallowing of large numbers of the microbes which cause disease. It is not only "wind" and "indigestion" which may be, and frequently are, thus set up; but diarrhœa, inflammations, and other most serious internal complaints. Let it also be preached broadcast by nurses, midwives, and doctors that even though a screaming baby can be reduced to silence by giving it a "comforter" to suck, yet it is quite easy to train young babies to "be good." The way to do this is to take no notice of them at all when they cry, provided always that any cause of actual discomfort be removed. Thus a baby may cry because its napkins are soiled, in which case they must of course be changed and the child thus made comfortable; or because it is hungry, in which case it should be fed provided its proper meal-time has arrived (but not otherwise). When, however, a child screams merely for the joy of it, so to speak, that child should be left severely alone; to pick it up and dandle it, to rock the cradle, or to push a "comforter" between its gums is simply training it into bad habits. Babies can be taught good habits just as easily as bad; and it is surprising how quickly they do pick them up, if mothers will only exercise patience and sacrifice their peace for a few days to begin with.

### Ear and Teeth Diseases.

The principle of infant management thus roughly sketched provides plenty of ground for the absolute condemnation and rejection of the "comforter." But the evils of this still popular engine of infant morbidity are by no means exhausted in the catalogue already given. Recent writers have proved that other serious and common diseases of childhood can be traced in a great many cases to the unclean thing. Mr. W. H. Bowen has explained * how ear disease, often permanently affecting the hearing and sometimes even endangering life, is caused by the deadly "comforter." Dr. E. H. R. Harries, in the same way, devotes an article† to its influence in setting up decay of the teeth. He published in that article a series of drawings showing the most horrible dental deformities directly traceable to the perpetual habit of "comforter" sucking. It is notable that, as a fever hospital officer, he met with these cases in children suffering from diphtheria, scarlet fever, etc.; and it is not at all improbable that the germs of those diseases found access to the throats of the patients by means of the very "comforter" which had already ruined the teeth.

Dr. Harries is no fanatic on the subject, and it must not be supposed that he is a faddist on this matter. He expressly states that he does not attribute all decayed teeth in children to the use of "comforters," or that all children who have them will necessarily get the dreadful mouths shown in his drawings; but he does produce good evidence that in the selected cases he describes the one thing had led to the other, and to the detriment of the interests of the child. The researches of school medical officers all over the kingdom have revealed the appalling condition of the teeth among the children of the poorer classes; and it is not at all impossible that the universal "comforter" may explain a considerable part of these decayed teeth.

The lessons to be learnt from these examples of the far-reaching and disastrous consequences of the "comforter" habit reinforce the above objections.

* *The Midwife's Pronouncing Dictionary.* The Scientific Press, Ltd. 1s. net.

* *Lancet*, Sept. 9, 1911, p. 758.
† *Ibid.* Nov. 11, 1911, p. 132.

